# Development of a Tailored Intervention With Computerized Clinical Decision Support to Improve Quality of Care for Patients With Knee Osteoarthritis: Multi-Method Study

**DOI:** 10.2196/resprot.9927

**Published:** 2018-06-11

**Authors:** Stijn Van de Velde, Tiina Kortteisto, David Spitaels, Gro Jamtvedt, Pavel Roshanov, Ilkka Kunnamo, Bert Aertgeerts, Per Olav Vandvik, Signe Flottorp

**Affiliations:** ^1^ Centre for Informed Health Choices Norwegian Institute of Public Health Oslo Norway; ^2^ Department of Internal Medicine Tampere University Hospital Tampere Finland; ^3^ Department of Public Health and Primary Care Katholieke Universiteit Leuven Leuven Belgium; ^4^ Oslo and Akershus University College of Applied Sciences Oslo Norway; ^5^ Department of Medicine McMaster University Hamilton, ON Canada; ^6^ Duodecim, Scientific Society of Finnish Physicians Helsinki Finland; ^7^ Institute of Health and Society University of Oslo Oslo Norway; ^8^ Making GRADE the Irresistible Choice (MAGIC) Oslo Norway

**Keywords:** decision support systems, clinical, practice guidelines as topic, guideline adherence, evidence-based medicine, osteoarthritis, knee, focus groups

## Abstract

**Background:**

Clinical practice patterns greatly diverge from evidence-based recommendations to manage knee osteoarthritis conservatively before resorting to surgery.

**Objective:**

This study aimed to tailor a guideline-based computerized decision support (CDS) intervention that facilitates the conservative management of knee osteoarthritis.

**Methods:**

Experts with backgrounds in clinical medicine, research, implementation, or health informatics suggested the most important recommendations for implementation, how to develop an implementation strategy, and how to form the CDS algorithms. In 6 focus group sessions, 8 general practitioners and 22 patients from Norway, Belgium, and Finland discussed the suggested CDS intervention and identified factors that would be most critical for the success of the intervention. The focus group moderators used the GUideline Implementation with DEcision Support checklist, which we developed to support consideration of CDS success factors.

**Results:**

The experts prioritized 9 out of 22 recommendations for implementation. We formed the concept for 6 CDS algorithms to support implementation of these recommendations. The focus group suggested 59 unique factors that could affect the success of the presented CDS intervention. Five factors (out of the 59) were prioritized by focus group participants in every country, including the perceived potential to address the information needs of both patients and general practitioners; the credibility of CDS information; the timing of CDS for patients; and the need for personal dialogue about CDS between the general practitioner and the patient.

**Conclusions:**

The focus group participants supported the CDS intervention as a tool to improve the quality of care for patients with knee osteoarthritis through shared, evidence-based decision making. We aim to develop and implement the CDS based on these study results. Future research should address optimal ways to (1) provide patient-directed CDS, (2) enable more patient-specific CDS within the context of patient complexity, and (3) maintain user engagement with CDS over time.

## Introduction

### Background

Computerized decision support (CDS) is a technology that uses patient data to provide relevant medical knowledge when needed; it may improve adherence to evidence-based recommendations [1-3]. It can also target patients to facilitate shared decision making and to empower and motivate them [4-6]. Unfortunately, CDS is a complex intervention that has not consistently delivered positive returns on substantial investment [7-11]. Although multiple systematic reviews have provided some insights about these factors, we are only beginning to understand how to use CDS to improve care processes and patient outcomes [7,9,12-14].

We undertook the GUideline Implementation with DEcision Support (GUIDES) project to (1) investigate the factors that determine successful CDS implementation, (2) develop a checklist to address these factors, (3) develop a tailored CDS intervention to improve care, and (4) plan a multicountry cluster randomized controlled trial to assess the effectiveness of that intervention. This paper describes the methods and results for objective 3.

We chose CDS for knee osteoarthritis as a target medical condition for several reasons. The lifetime prevalence risk of symptomatic knee osteoarthritis is 45% [15] and is projected to increase with the rise of obesity and an aging population [3]. The guidelines for this condition largely agree on conservative management, including pharmacological and nonpharmacological interventions (eg, exercise, weight loss for overweight and obese patients) [16,17]. However, guideline adherence by health care professionals is remarkably low. A systematic review of studies assessing appropriateness of care found that only 36% of the eligible knee osteoarthritis patients receive the recommended nondrug treatment and 38% receive the recommended drug treatment in high-income countries [18]. This is opposed to a quality indicator score of nearly 80% for surgical referral. Although joint replacement surgery is effective, it is associated with significant perioperative complications, postoperative pain and functional limitation, and costs [19]. The number of joint replacements and the need for reoperations may be reduced if conservative modalities were exhausted first [20]. Knee arthroscopy is another frequently performed surgical procedure, despite guidelines with strong recommendations against its use [21].

Tailored implementation interventions are strategies that are designed to achieve desired changes in health care practice based on an assessment of determinants of health care practice [22]. Such strategies can include a single improvement intervention or they can be multifaceted. Determinants are factors that might prevent or enable adherence and these may relate to the health care professional, the patient, and the given context [23]. Tailored implementation can be used to improve care for different medical conditions and types of care practices. A Cochrane systematic review provides evidence of the benefits of tailored implementation, but it remains unclear how best to tailor interventions [24]. In this paper, we describe our methods, processes, and experiences to contribute to learning for how to develop CDS interventions that require bridges between multiple research fields [25,26].

### Objectives

The objective of this study was to determine how to tailor a CDS intervention for general practitioners (GPs) and patients that facilitates the conservative management of patients with knee osteoarthritis in Norway, Belgium, and Finland as defined by evidence-based recommendations [16,17].

## Methods

### Study Design

We collected input from 9 experts overall (with backgrounds in clinical medicine, research, implementation, or health informatics) and from 8 GPs and 22 patients coming from Norway, Belgium, and Finland. The choice of countries was pragmatic.

We tailored the intervention in 4 steps: (1) selection of the most important recommendations for implementation, (2) development of an implementation strategy, (3) forming the CDS intervention concept, and (4) identification of determinants that may affect the success of the suggested CDS strategy.

On the basis of the experience within the author group, we anticipated that a CDS intervention would be among the selected strategies in step 2 or that CDS at least would be able to facilitate other strategies.

We developed worksheets to provide support for steps 1 to 3 (see Multimedia Appendix 1). Eight experts of whom 4 were authors of this paper (SVDV, SF, GJ, and DS) used the worksheets to provide their considered judgment. We organized 6 focus groups, 2 in each country, to obtain input for step 4. The number of focus groups was a pragmatic choice. We discussed the results of each step within the project group and made decisions in consensus. We reported the focus group methods in accordance with agreed standards [27].

The project built on the results of the European Union (EU)–funded Tailored Implementation for Chronic Diseases project [22,23]. The author group included experts with a strong commitment to evidence-based medicine and broad expertise related to the clinical care of patients with knee osteoarthritis, and to the development, implementation, and evaluation of CDS [7,28-33].

### Step 1: Selection of Recommendations

We identified evidence-based recommendations for the conservative management of knee osteoarthritis from existing overviews of guidelines [17,34]. Eight experts prioritized the most important recommendations for implementation by assessing each recommendation for the following questions (worksheet A in Multimedia Appendix 1):

1. Is the recommendation feasible for practice?
2. Is adherence to the recommendation important?
3. Is there a large amount of inappropriate practice for this recommendation?

We only retained those recommendations where at least three-fourth of the experts agreed that it should be prioritized.

We also extracted information on the strength of recommendations, when this was presented according to the Grades of Recommendation Assessment, Development, and Evaluation (GRADE) approach [35]. We agreed beforehand to consider both strong and conditional recommendations.

### Step 2: Development of an Implementation Strategy

We identified published qualitative evidence syntheses to take account of the results of research on determinants of adherence to knee osteoarthritis recommendations [36-39]. For each determinant, 4 experts considered if it related to a specific recommendation or to all the recommendations, and they used worksheet B (Multimedia Appendix 1) to assess the following:

1. Is the determinant likely to have an important impact on adherence to the recommendation?
2. What would be a potential implementation strategy that takes into account this determinant?
3. Is the strategy likely to have an important impact on improving adherence?
4. Is the strategy feasible to implement?

The assessment of the determinants could lead to the selection of a single-faceted implementation strategy or to opting for a multifaceted package of implementation strategies.

### Step 3: Forming the Computerized Decision Support Intervention Concept

We formed the concept of a CDS intervention to provide support in the electronic medical record (EMR). The CDS was intended to be operationalized with the Evidence-Based Medicine Electronic Decision Support System (EBMeDS; by Duodecim Medical Publications Ltd). EBMeDS receives structured patient data from EMRs and returns CDS based on programmed algorithms [30,40]. Computer scripts check relevant patient data in relation to predefined algorithms to determine if it would be appropriate to present a given recommendation. EBMeDS can be linked to recommendations that are presented in the Making GRADE the Irresistible Choice (MAGIC) authoring and publication platform—an electronic platform for point-of-care evidence summaries and decision aids [6,28]. We chose to use EBMeDS and MAGICapp based on previous collaborations in research and implementation projects.

For each selected recommendation, we conceived a CDS algorithm. Five experts used worksheet C (Multimedia Appendix 1) to assess if it was appropriate to apply the suggested algorithms by using the following questions:

1. Is appropriate operationalization with CDS likely for this algorithm?
2. Is appropriate user response likely for this algorithm?

We did not use any majority thresholds for the selection of algorithms in this step.

### Step 4: Focus Groups on Determinants of an Effective Computerized Decision Support Strategy for Knee Osteoarthritis

**Figure 1:**
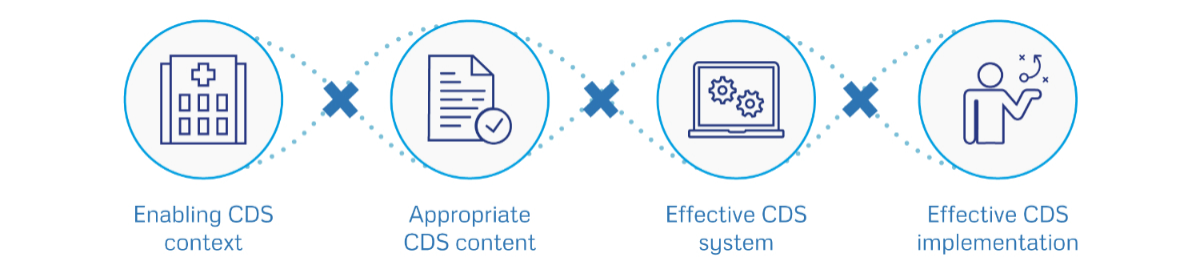
The GUideline Implementation with DEcision Support (GUIDES) checklist contains 16 factors covered by 4 domains that potentially impact on the success of computerized decision support (CDS) to implement recommendations. The CDS context domain focuses on the circumstances in which CDS can be potentially successful; the CDS content domain focuses on the factors shaping the success of the advice produced by the CDS system; the CDS system domain focuses on the features belonging to the CDS tool; and the CDS implementation domain refers to the factors affecting the CDS integration in practice settings.

The focus groups covered the suggested CDS for the selected recommendations, the factors that determine successful use of CDS, and selection of the most important factors. During the focus group, we presented a hypothetical case of a patient with knee osteoarthritis, and we used screenshots to illustrate the suggested CDS strategy. We first identified determinants of successful CDS through brainstorming. When no additional factors were suggested by the participants, the moderator used the GUIDES checklist (Figure 1) to ask probing questions on factors that were not yet discussed [41,42]. A detailed interview guide is available in Multimedia Appendix 2. We tested the interview guide on colleagues before the start of the actual focus groups.

We aimed to recruit 3 GPs and 3 knee osteoarthritis patients per focus group. We used convenience sampling based on our personal networks and recommendations from colleagues. We included patients from different age groups, with different degrees of osteoarthritis and patients having osteoarthritis as a single condition together with patients having comorbidities. We ensured that patients and GPs in the same group did not have a personal doctor-patient relation. We also ensured that there were at least as many patients as GPs to increase patients’ confidence when expressing views and experiences.

We contacted every participant before the focus group to collect informed consent and to address any questions. The focus groups took place in meeting rooms of the participating institutions.

SVDV (male) moderated the focus groups in Norway and Belgium and TK (female) in Finland. TK was experienced in conducting focus groups, and SVDV received training beforehand [30,32]. The moderators emphasized that both positive and negative feedback about the CDS intervention was important. We audio-recorded each focus group and an observer took notes. We transcribed key parts of the focus groups, but we did not do a full transcription of the recordings.

### Data Analysis

We transcribed the data from the focus groups anonymously and applied the framework analysis approach [43]. One researcher (SVDV) analyzed the transcribed parts of the interview recordings and used an Excel worksheet to extract all quotes, including determinants and alternative or additional strategies for the suggested CDS intervention that the participants mentioned in step 4. We classified the data according to the GUIDES checklist (Multimedia Appendix 3). If we could not link quotes to a specific field in the chosen checklist, we categorized this as general. SVDV evaluated which quotes were related to others in order to group them. We then labeled and analyzed the quotes as such. For every labeled item, we explored if it was linked to the focus group procedure (without GUIDES checklist through brainstorming vs through a structured discussion based on the GUIDES checklist). One researcher (SF) double-checked the grouping, labeling, and analysis. Three researchers who participated in the focus groups (SF, TK, and DS) double-checked the reporting of the interviews. The researchers resolved disagreement by consensus.

### Ethics Approval and Consent

The Regional Committee for Medical Research Ethics in South East Norway and the University Hospitals Leuven Medical Ethics Committee in Belgium waived the requirement to seek ethical approval. In Finland, approval for the study was received from the Ethics Committee of the Pirkanmaa Hospital District.

### Availability of Data and Material

All data generated or analyzed during this study are included in this published paper and its Multimedia Appendix files.

## Results

### Selection of Recommendations, Implementation Strategy, and Potential Computerized Decision Support Intervention

We selected 9 recommendations (Textbox 1) out of 22 recommendations that we extracted from the overviews of knee osteoarthritis guidelines. Multimedia Appendix 4 provides details on the ratings for every recommendation. The Finnish experts did not prioritize the recommendation on clinical diagnosis, as x-ray is a usual part of the diagnostic assessment if long-term treatment is required.

Two guidelines graded their recommendations according to GRADE, and we extracted information on the strength of the recommendations based on these guidelines [21,44]. Five prioritized recommendations were strong recommendations, 3 were conditional recommendations, and no strength was currently available for the diagnostic recommendation.

We extracted 30 factors that might affect adherence to knee osteoarthritis recommendations from 4 qualitative evidence syntheses [36-39]. They were categorized in 4 domains: guideline factors, health professional factors, patient factors, and incentives and resource factors [23]. Guideline factors addressed clarity, specificity, and ease of implementation of recommendations. Key themes for health professional factors were personal opinions and attitudes about the importance of knee osteoarthritis and its progression and management. Another factor is the information needs of health care professionals about recommended practice. Patient factors related to information needs; shared decision making; and the role of patient opinion, motivation, and behavior.

Both health professional and patient factors contributed to obtaining a timely diagnosis. Incentives and resources factors included financial incentives and disincentives to adherence and the limited time available during a patient consultation. The experts assessed these determinants and confirmed that a guideline-based CDS strategy was appropriate, combined with a need for health care provider education, the availability of patient information, and strategies to support patients in realizing lifestyle changes. We did not consider actions to make health system changes because we could not take direct responsibility for this and judged that such changes were not feasible in the context of our project.

**Overview of the prioritized recommendations for knee osteoarthritis.**

- A clinical assessment is sufficient to diagnose knee osteoarthritis [45]: no grade of recommendation available (NA)
- Patients with knee osteoarthritis should receive self-management information and education [17]: conditional recommendation (CR)
- Patients who are overweight should be encouraged to lose weight [17]: strong recommendation (SR)
- Low-impact aerobic exercise (land or water based) should be recommended to patients [17]: SR
- Cardiovascular or strengthening exercises should be recommended to patients [17]: SR
- Oral nonsteroidal anti-inflammatory drug (NSAID) should only be used after acetaminophen [17]: CR
- Gastroprotection for high-risk patients [17]: SR
- Topical NSAID should be used as adjunctive and alternative to oral agents [17]: CR
- Arthroscopy with debridement is not recommended for the management of symptomatic knee osteoarthritis [17]: SR

We formed the concept for 6 algorithm-based CDS scripts to support implementation of the prioritized recommendations:

1. A first script suggests to the GP to consider if the diagnosis of knee osteoarthritis is relevant in patients aged above 45 years with a knee complaint code registered in the EMR, and it presents the diagnostic criteria.
2. Another script suggests discussing the treatment plan for patients with a knee osteoarthritis diagnosis and to provide patient information. This reminder links to patient information and patient decision aids that provide detailed information on the benefits and harms of every treatment option and the related practical issues.
3. For every knee osteoarthritis patient, a reminder shows that exercise is recommended.
4. In patients that are overweight or obese, a reminder suggests dietary counseling and bariatric surgery if the body mass index (BMI) is above 35 kg/m^2^.
5. If the BMI value for a patient with knee osteoarthritis is missing or when its calculation is older than 2 years, a reminder suggests adding the missing clinical data in the EMR.
6. The last script generates a reminder in patients with a prescription for oral NSAIDs to consider topical NSAIDs and/or paracetamol.

**Figure 2:**
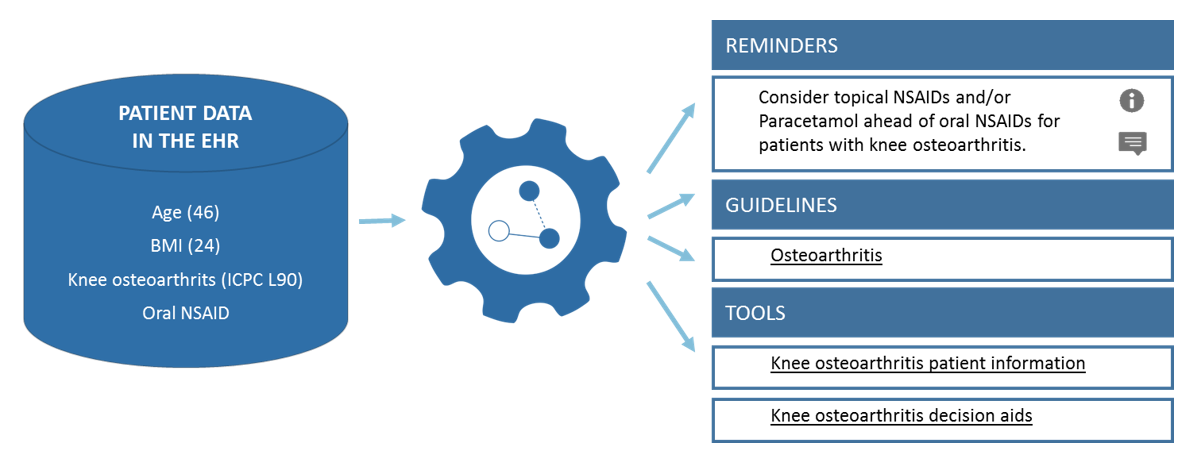
Illustration of the generated computerized decision support (CDS) in the style of Evidence-Based Medicine Electronic Decision Support System (EBMeDS) after having entered the body mass index (BMI) and after having prescribed an oral nonsteroidal anti-inflammatory drug (NSAID). On the basis of the patient data in the electronic record, the CDS system presents patient-specific reminders, links to guidelines, and practical tools. When clicking on the reminder, the general practitioner (GP) receives more information about the recommendation, how this reminder works, and on which guideline(s) the information is based. ICPC: International Classification of Primary Care, second edition.

**Figure 3:**
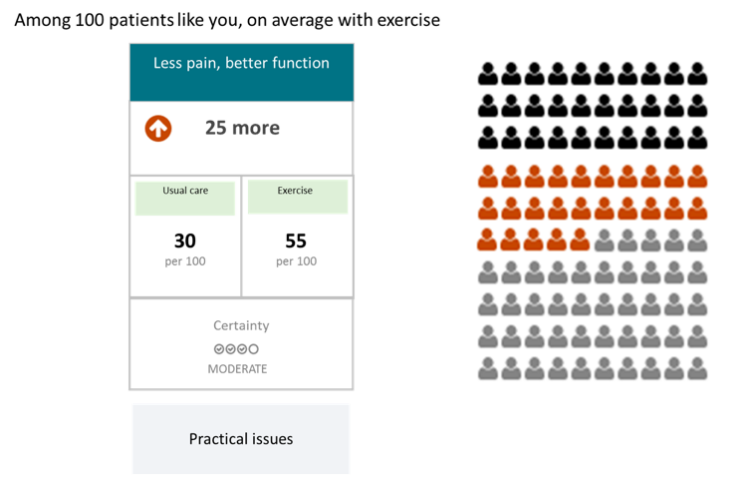
Illustration of the decision aid on exercise in the style of MAGICapp. The general practitioners (GPs) and the patient can use this to discuss the benefits and practical issues for potential treatment options. The decision aid provides graphic displays of the benefits and harms of different treatment options together with information about certainty in estimates of effect according to Grades of Recommendation Assessment, Development, and Evaluation (GRADE). When clicking on the “Practical issues” button, another information layer opens with additional information (eg, description of the procedure, adverse effects).

Figures 2 and 3 provide an illustration for parts of the CDS and the consultation decision aids. Multimedia Appendix 2 provides further illustrations.

### Determinants Affecting Success of the Suggested Computerized Decision Support Strategy for Knee Osteoarthritis

We conducted 6 focus groups (2 in each country). A total of 22 patients and 8 GPs participated in the focus groups. In Finland, all the participants were patients. No participants dropped out. Moreover, 19 patient participants were females, and only 3 were males. Their age ranged from 26 to 85 years. GP participants were mainly male, and only 1 was female. Age ranged from 29 to 69 years.

The participants suggested 211 factors that might affect the success of the presented CDS intervention. When we combined the factors that are related or somewhat related, we ended up with 59 unique factors. Of the unique factors, 14% (8/59) were identified by patients only, 39% (23/59) by GPs only, and 47% (28/59) by GPs in 1 group and by patients in another group. The median number of unique factors suggested per focus group was 31 (range 15-37).

The participants selected 47 factors that they considered most important. Nine factors were discussed in each country, among which 5 factors were also prioritized in each country (see Textbox 2). We grouped the factors in 7 categories that we describe in detail below. Multimedia Appendix 5 lists all the suggested factors and indicates if the factors were prioritized and if they were related to one or more countries.

#### Factors Related to Information Needs for Patients

Participants thought that patient-directed CDS could be a good strategy by providing reliable information directly to patients. Some found that it is particularly useful, given the time limitations of a consultation, and that it could reduce unwarranted delays when ordering consultations. Both patients and GPs mentioned that informing patients better can increase the potential for shared decision making:

Patients need direct access to CDS so that they can prepare themselves for a consultation.

Patient, Norway

It is an advantage when reliable information can be sent to the patient, because GPs often have to use time to reassure patients that have read inappropriate information from unreliable sources.

GP, Belgium

Several participants preferred that the GP would act as an intermediate recipient of the patient-directed CDS:

An alternative approach is that the GP gets an overview of the CDS for the patient and then decides if it is relevant to forward it to the patient.

GP, Norway

Other patients might prefer not to receive any CDS information at all. This might be influenced by previous experiences with information technology. GPs do not always know if a patient wants additional information, and posters or flyers in the waiting room could inform patients that they can ask their GP for patient information.

Participants mentioned some potentially negative consequences of providing CDS directly to patients, including anxiety, inappropriate management, and the risk that CDS might replace the personal contact with the GP. Multiple GPs and patients preferred to deliver CDS for patients during the consultation so that the GP can advise the patient in person:

GPs should assess the CDS and give treatment advice that is suitable for that patient.

Patient, Finland

Patients recognized the importance of knowledge about what to do, but mentioned other barriers. Personal beliefs and desires about tests or treatments can be strong. Patients may need support to help achieve lifestyle changes, and some gave the example of physiotherapy for patients that are less motivated. Another patient expressed the need for more practical information:

More information for patient is needed about how to live with osteoarthritis. CDS could inform about osteoarthritis schools for patients and patient support groups.

Patient, Norway

#### Factors Related to Information Needs for General Practitioners

CDS could help GPs not to forget certain treatment options and to stay updated about new or changed recommendations. A patient suggested that the CDS intervention could make GPs more attentive to osteoarthritis. One GP mentioned that CDS should not limit the treatment choices of GPs. Furthermore, CDS should inform and alert in a constructive way but not criticize the GP:

It is obvious that all GPs do not know all treatment options to all diseases, so CDS could help them.

Patient, Finland

**Nine factors (grouped into themes) that may affect success for the suggested computerized decision support (CDS) strategy for knee osteoarthritis and that general practitioners (GPs) and patients suggested in Norway, Finland, and Belgium. Five factors were prioritized in each of the 3 countries.**

*Patient needs*

- Potential to address the information needs and demands for patients *(prioritized)*
- Acceptability of CDS for patients
- Need for personal dialogue about the CDS between the GP and patient *(prioritized)*

*GP needs*

- Potential to address the information needs for GPs *(prioritized)*

*Patient data*

- Accuracy and completeness of the available patient data in the electronic medical record (EMR)
- Benefits of CDS on quality of patient data in the EMR

*Workflow and workload*

- No common factors for the 3 countries

*CDS content*

- Credibility of the CDS information *(prioritized)*

*CDS system*

- Effort required to use the CDS system
- Timing of delivery of CDS for patients *(prioritized)*

*CDS implementation*

- No common factors for the 3 countries

GPs asked if CDS could help them to identify patients that are coping badly with their disease. This would make it possible to devote extra attention to those patients that need it most.

A patient commented on the limitations of CDS:

Technology alone is not enough, a lot has to do with the personal contact between the patient and the physician, and the physician needs to know the patient’s perspective.

Patient, Norway

Several patients suggested that other health care professionals should also receive CDS.

#### Factors Related to Patient Data

GPs mentioned that the patient’s EMR could have data gaps. Often, GPs record symptoms instead of a diagnosis. Some GPs found it positive that CDS could identify and help filling gaps in the patient’s record. Some said that CDS would motivate them to improve the quality of their EMR. Another GP mentioned that requests to register extra data (such as BMI) should be limited:

Physicians should only be asked to enter patient data when this is having a positive impact on the patient outcome.

GP, Norway

Now I have to bring my medical data on a paper to the GP or occupational therapist.

Patient, Finland

Both patients and professionals discussed that additional patient data are needed to allow good CDS. Data on patient adherence and effect of the treatment were mentioned several times. GPs also suggested registering the diagnosis or complaint for the encounter. Knowledge about the reason for encounter could help GPs to prepare for the consultation and could prevent the CDS from generating information that is irrelevant for the reason for the encounter:

It would be interesting if the GP could indicate the reason for the encounter and that CDS is triggered accordingly.

GP, Norway

#### Factors Related to Workflow and Workload

The limited time to use and discuss the CDS during a patient encounter is a barrier, especially when patients want to discuss multiple problems. Therefore, CDS needs to be well integrated. Given the time pressure, CDS can create stress for GPs. It may be necessary to plan an additional consultation to discuss the information given by the CDS.

CDS could also save time for GPs if it facilitates fast information retrieval. Some GPs mentioned that it is faster to use CDS than to find information in a book:

CDS should fit in the workflow so that it has no negative impact on the amount of patients seen by the clinician.

GP, Belgium

A challenge for timely CDS is that the GPs often do not enter the diagnosis for new complaints directly in the EMR. A risk with CDS is that it might disturb the personal contact between the patient and the GP.

#### Factors Related to the Computerized Decision Support Content

CDS should recommend specific action. Many participants requested more information about which type of exercise works best:

I got the instruction to bike 30 minutes/day. It has never been clear why I had to do exactly this training. Is this type of training more beneficial than others?

Patient, Norway

A GP mentioned that CDS should provide nuances that are specific to a patient and that this can be too big of a challenge for some problems. It might only be possible to provide CDS if the condition and the recommended action can be defined in sufficient detail.

Some GPs emphasized that CDS has to be based on evidence-based guidelines that are up to date. The participants perceived the presented CDS intervention as a reliable tool. The certainty of the evidence should be clear for every CDS recommendation. Some GPs found detailed information about the treatment effect important, whereas other GPs considered this information as trivial facts. Several participants considered it a limitation that the CDS presents mean treatment effects, when the effect that an individual patient experiences can be different from these mean effects.

GPs commented that CDS should be relevant for the patient’s problem and that irrelevant CDS content can be disturbing:

CDS can diverge the focus of the consultation to the topics suggested by the CDS instead of the problem raised by the patient.

GP, Norway

Insight in how the CDS is triggered is desirable in case GPs have doubts about the CDS.

Those that implement CDS should carefully reflect over the amount of CDS, because too much information can overload both patients and GPs:

When the information becomes too much, then you lose focus.

GP, Norway

The CDS should not overload the patient, too much information will lead to forgetting parts of it.

GP, Belgium

Both patients and GPs suggested to divide CDS over time, for example, over different consultations for the same patient. In the case of patients with comorbidities, the CDS system should prioritize which content is most important. A GP commented that CDS should cover a minimum number of potential patient problems:

CDS should at least cover 100 to 200 diagnoses before it becomes interesting to use.

GP, Norway

#### Factors Related to the Computerized Decision Support System

CDS should be easy to use. The system should work fast and with minimal data traffic. One GP noticed that experience with the system might reduce the time required to use it:

Within the EMR, physicians already need to click a lot. CDS requires additional clicks and I don't know if I am motivated to make that additional effort.

GP, Belgium

GPs desired CDS that is short and immediately understandable. Some GPs suggested a multilayered approach where it is possible to click for further information. Several GPs emphasized the important role of a visual display that includes illustrations.

Patients suggested multiple channels to deliver patient-directed CDS:

CDS should appear in all the communication channels that a patient uses. For example a smartphone, e-mailbox, etc.

Patient, Finland

Patients do not have access to CDS that is presented in the EMR. Can the electronic patient record be an instrument to provide CDS to patients?

Patient, Norway

GPs mentioned that they should have control over the system, including the potential to customize which CDS they will receive and the option to receive CDS only on demand. Other GPs preferred CDS that is provided automatically but not as pop-ups. GPs expressed different preferences regarding the timing of the CDS. Some found CDS most effective during the consultation, whereas others would read CDS before the patient encounter if they knew the contact reason.

A challenge is that the GPs over time might be less interested in the CDS information:

After a while you will no longer give attention to the information that you have read several times before. This includes the risk that you do not notice that new information is available.

GP, Belgium

#### Factors Related to the Computerized Decision Support Implementation

Participants mentioned that the CDS must be intuitive, but the GPs and patients should always receive information about the system beforehand. Some GPs also requested training, even for intuitive systems. Those responsible for implementing the CDS system should market the CDS with clear examples of the advantage of CDS. One GP suggested marketing CDS toward patients, so that patients would ask if their GP is using such a system:

The system should be marketed and the best strategy is to demonstrate success through the involvement of superusers or through demonstration in pilots.

GP, Norway

Participants discussed the need to monitor system performance and referred to other eHealth initiatives with adequate electronic feedback channels. Sufficient technical support and budget is needed. Participants suggested public governance of CDS. Some thought it could also be private but not financed by the drug industry:

The system should not be incomplete when it is implemented, because then it will not be a practical solution to the user; The system should be continuously improved.

Patients, Finland

## Discussion

### Principal Findings

The results of this study inform the development and implementation of a tailored CDS intervention. The experts prioritized 9 knee osteoarthritis recommendations for implementation. To implement these recommendations, we selected CDS combined with education for GPs, and patient information and support to achieve lifestyle changes. We formed the concept for this CDS intervention and discussed it with patients and GPs during focus groups. Both patients and GPs found that the strategy has potential to improve the quality of health care for patients with knee osteoarthritis. Use of the GUIDES checklist allowed us to identify additional factors that would otherwise have been missed by the focus group. These findings have been submitted for publication to *Implementation Science* (S Van de Velde, unpublished data, May 2018).

### Strengths and Limitations

We followed a systematic approach, including considered judgment by experts and focus groups with patients and GPs, to build knowledge that can inform the development of a tailored CDS intervention to improve the quality of care for patients with knee osteoarthritis [23,46].

Although it seems intuitive to tailor interventions to the determinants of practice, existing evidence indicates that we can only expect moderate effects on outcomes through tailored implementation [24,47]. Our systematic approach was also a lengthy and resource-intensive process. When embarking on a complex intervention, we consider it good practice to do this systematically, in line with guidance from the UK Medical Research Council on complex interventions [48]. This helps to ensure a greater return on investment and prevents unnecessary trial and error or unintended negative consequences.

We involved a broad range of stakeholders during the development of the intervention. We assume that this multiperspective approach allowed us to identify the diversity of factors. Some of the health care professionals and researchers had a professional relationship with the authors, but as our only interest was to improve the quality of care, we do not expect that this had an influence on the feedback.

Only 8 GPs participated in the focus groups compared with 22 patients. It was difficult in each of the participating countries to find GP participants for the focus groups. In Finland, only patients participated in the focus groups, as we were unable to recruit GPs there. Our comparison of the factors identified per country is by consequence incomplete.

Our multicountry approach increases the generalizability of the strategy. Most of the factors seem to apply to the 3 countries, and it is plausible that the CDS intervention can also be implemented in other countries. However, we did not systematically evaluate generalizability to other countries that may share less similarities than Norway, Belgium, and Finland.

### Implications

Any decision to use CDS, other interventions, or additional implementation strategies should be based on an assessment of the determinants of health care practice that affect whether the desired changes can be achieved [23]. Furthermore, it is important to be aware of the factors shaping CDS effectiveness [9]. This study has advanced the understanding of such determinants and CDS success factors.

We now aim to develop the CDS based on the input from the focus group discussions. We then plan to conduct a usability evaluation among the users and an evaluation of the accuracy of the CDS recommendations and the relevant patient data in the EMR [49-51]. We intend to evaluate this intervention in a multicountry cluster randomized controlled trial and assess its cost-effectiveness.

The evidence on the effect of CDS on patient outcomes is very uncertain, and only 1 trial has been conducted so far on patients with knee osteoarthritis. That trial studied the effect of CDS for GPs combined with a patient-directed intervention and found slightly better function and increased physical activity at 12 months but no differences for pain, depressive symptoms, and BMI [52].

Multiple key questions emerged from the focus groups. First, we do not know the best way to provide patient-directed CDS. Approaches previously used should be investigated within this context [53,54]. In addition, the field of CDS needs to engage on a discussion with the field of patient decision aids [55]. Second, it is not clear how best to collect and use additional patient data to enable more patient-specific CDS. Integrating evidence from reliable analyses of patient subgroups in randomized trials and systematic reviews may provide a reasonable starting point to making CDS more patient-specific [56,57]. Third, it is not clear how to maintain users’ interest and engagement with the CDS over time. CDS research needs to explore how sustainability can be achieved [58-60]. A systematic review of factors that improve long-term use of CDS may provide a starting point for this agenda.

### Conclusions

The focus group participants expressed their support for the CDS intervention as a tool to improve the quality of care and the outcomes for patients with knee osteoarthritis through shared, evidence-based decision making. GPs and patients perceived the strategy as helpful for their information needs. It might also improve the quality of patient data in the EMR. It is important that GPs can use the CDS with limited effort, and the usability of the CDS should be tested before full-scale implementation.

## Appendix

Multimedia Appendix 1 Worksheets.

Multimedia Appendix 2 Interview guide.

Multimedia Appendix 3 GUideline Implementation with DEcision Support (GUIDES) checklist.

Multimedia Appendix 4 Prioritization of recommendations.

Multimedia Appendix 5 Focus group data.

## Abbreviations

BMI: body mass index
CDS: computerized decision support
EBMeDS: Evidence-Based Medicine Electronic Decision Support System
EMR: electronic medical record
EU: European Union
GP: general practitioner
GRADE: Grades of Recommendation Assessment, Development, and Evaluation
GUIDES: GUideline Implementation with DEcision Support
MAGIC: Making GRADE the Irresistible Choice
NSAID: nonsteroidal anti-inflammatory drug
