# Supplementary material for: Development of a Tailored Intervention With Computerized Clinical Decision Support to Improve Quality of Care for Patients With Knee Osteoarthritis: Multi-Method Study
Source: JMIR Res Protoc. 2018 Jun 11;7(6):e154. doi: 10.2196/resprot.9927 (PMC6018233; doi:10.2196/resprot.9927)
Supplement: Multimedia Appendix 1 [file resprot_v7i6e154_app1.pdf]

# Additional file 1

## Worksheet A. Prioritisation of recommendations for implementation

| Recommendations | Is this recommendation feasible for practice? | Are the consequences of not following this recommendation important? | Is there a large amount of inappropriate practice for this recommendation? | Is implementing this recommendation a priority? |
|---|---|---|---|---|
| | Consider:<br><br>Presence of barriers or need for efforts to implement the recommended practice. | Consider:<br><br>• Magnitude of intervention effect<br>• Risks associated with the intervention<br>• Level of evidence<br>• Balance of benefits and cost. | Consider:<br><br>• Evidence of underuse, misuse and/or overuse<br>• Evidence of avoidable health inequities. | Consider:<br><br>All the previous ratings to assess if implementation of the recommendation is a priority. |
| Scoring system: 1= Strongly disagree, 2= Disagree, 3= Disagree and agree equally, 4= Agree, 5= Strongly Agree, N= No opinion.<br>Comments can be included in each cell following the score. | | | | |
| **1.** | | | | |
| **2.** | | | | |
| **3.** | | | | |

Worksheet A is adapted from the worksheets developed in the Tailored Implementation for Chronic Diseases project (TICD).

# Worksheet B. Selection of an implementation strategy

| **Determinant and recommendation(s) to which it applies** | **Is this determinant likely to have an important impact?** | **What would be a potential implementation strategy?** | **Is this strategy likely to have an important impact?** | **Is this strategy feasible to implement?** | **Should the strategy be targeted?** |
|---|---|---|---|---|---|
| Consider:<br><br>Information from brainstorming, literature reviews, surveys, interviews, focus groups, routinely collected data, observation.* | Consider:<br><br>The importance of the determinant for adherence to the recommendations for the targeted problem. | Consider:<br><br>There may be more than one potential implementation strategy per barrier. | Consider:<br><br>The likely effectiveness of the implementation strategy in relation to the prioritised recommendations. | Consider:<br><br>The availability of the required human, technical and financial resources. | Consider:<br><br>All the previous ratings to assess if this strategy should be targeted. |
| Scoring system: 1= Strongly disagree, 2= Disagree, 3= Disagree and agree equally, 4= Agree, 5= Strongly Agree, N= No opinion.<br>Comments can be included in each cell following the score. | | | | | |
| **1.** | | | | | |
| **2.** | | | | | |
| **3.** | | | | | |

* Consult the TICD checklist for a comprehensive overview of potential determinants.
Worksheet B is adapted from the worksheets developed in the Tailored Implementation for Chronic Diseases project (TICD).

# Worksheet C. Appropriateness of CDS advice for specific recommendations

| **Suggested CDS advice and recommendation(s) to which it applies** | **Which structured patient data are required for CDS?** | **Is appropriate operationalisation with CDS likely?** | **Is appropriate user response likely?** | **Is this CDS advice appropriate?** |
|---|---|---|---|---|
| Consider:<br><br>What, for whom, when, where and how? | Consider:<br><br>Cases in which the support should or should not be triggered. | Consider:<br><br>• If the condition and recommended action are defined in sufficient detail to be operationalised<br>• Completeness, accuracy and specificity of available patient data<br>• Likelihood of inaccurate CDS. | Consider:<br><br>• Evidence on the effect of CDS for the clinical setting, clinical task, type of outcome<br>• Rates of ignoring or overriding CDS for similar objectives<br>• Risks of errors because of CDS. | Consider:<br><br>All the previous ratings to assess if this suggested CDS advice is appropriate. |
| Scoring system: 1= Strongly disagree, 2= Disagree, 3= Disagree and agree equally, 4= Agree, 5= Strongly Agree, N= No opinion.<br>Comments can be included in each cell following the score. | | | | |
| **1.** | | | | |
| **2.** | | | | |
| **3.** | | | | |
