# Supplementary material for: Development of a Tailored Intervention With Computerized Clinical Decision Support to Improve Quality of Care for Patients With Knee Osteoarthritis: Multi-Method Study
Source: JMIR Res Protoc. 2018 Jun 11;7(6):e154. doi: 10.2196/resprot.9927 (PMC6018233; doi:10.2196/resprot.9927)
Supplement: Multimedia Appendix 2 [file resprot_v7i6e154_app2.pdf]

Additional file 2

Focus group interview guide

The focus groups have a maximum duration of 2 hours, including a 10 minutes break. The interviews are divided in an introduction (15`), a structured discussion (100`) and a conclusion (5`).

Introduction
(15´) The moderator welcomes the participants, reviews the agenda and describes the background, objectives and rules of the focus group.

- Background: The moderator introduces knee osteoarthritis as a common musculoskeletal disease with an important burden and summarises why quality improvement efforts are needed for this condition.
- Objectives: The moderator states that this focus group is intended to inform the development of an effective computerised clinical decision support system intervention to support the implementation of selected evidence-based recommendations for knee osteoarthritis. The moderator makes it clear that there are different strategies that might improve the quality of care for knee osteoarthritis, but that we will only focus on computerised clinical decision support systems. The moderator explains how decision support systems function and asks the participants if there are any questions.
- Focus group rules: The rules include that the use of jargon by professional participants will be discouraged, that there are no wrong or right answers, that both professional and patient participants can make essential contributions and that it is important to express both positive and negative views.

The moderator provides room for questions and comments. Afterwards the interview phase of the focus group is started and the recording of the discussion is activated.

Interview phase

- (15´) The moderator presents a hypothetical case of a patient with knee osteoarthritis and gives examples of potential computerised decision support according to evidence-based recommendations for this patient. The moderator asks if there are any general comments about the presented cases and the decision support.
- (30´) The moderator invites the participants to reflect over the factors that determine successful or unsuccessful use of computerised decision support systems. Next, the factors are discussed collectively and the moderator writes the suggested factors on a whiteboard. When all the factors have been listed, the moderator proposes a short break.
- (10´) Break
- (30´) The moderator explains that he will now ask probing questions to identify any factors that were not discussed. The probing questions are derived from the GUIDES checklist. The probing questions are limited to those factors that are not yet listed on the whiteboard. Examples of probing questions are:
    - Is the quality of the patient data sufficient to use CDS?

    - Do you think that the decision support comes at the right time?
    - What do you think about the display of the decision support?
    - Would there be any important information and training needs for the GPs to use the system?
- Additional factors are added to the whiteboard in another colour.
- (15´) Next, the participants are invited to reflect individually over which five factors are the most important determinants for successful use of computerised decision support systems and to rank them in order of importance. The participants receive numbered stickers that they can stick next to the factors that are listed on the flip-chart.

Conclusion

(5´) The moderator thanks the participants for their contributions and elaborates on the next steps in the project. The participants are reminded on how the information from the focus groups will be processed and that they can withdraw at any time from the study without giving a reason.

Patient case and suggested CDS intervention

A 46 year old male presents to his family doctor because of a painful knee. One year ago, he visited his doctor for the same complaint. Then the doctor diagnosed it as wear and tear and proposed paracetamol painkillers. Over the past weeks, the patient experiences an increasing pain in his knee, which is not alleviated sufficiently by the painkillers.

The computerised decision support system uses the date on age and the previously recorded knee symptom data and provides a reminder for the doctor (figure 1). This reminder suggests to consider the diagnosis knee osteoarthritis.

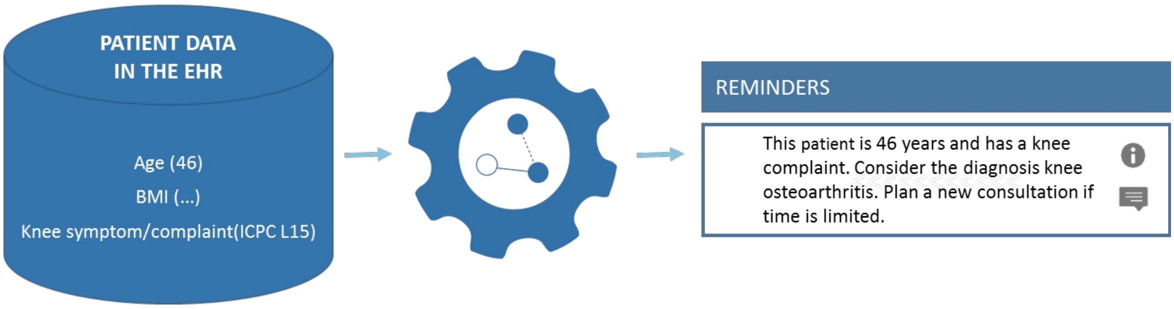

Figure 1: Illustration of the computerised decision support for the patient case in the style of EBMeDS. The decision support reminder to consider knee osteoarthritis as a diagnosis will also show up during a consultation for another complaint. Therefore it suggests to plan a new consultation if this is necessary. It is possible for the doctor to deactivate specific reminders.

When clicking on the reminder, the doctor can read more information on the diagnostic criteria. In another layer of information, more information is available on how this reminder works and on which guidelines the information is based (figure 2). In this situation the guideline is based on a recommendation from the National Clinical Guideline Centre in the UK.[1]

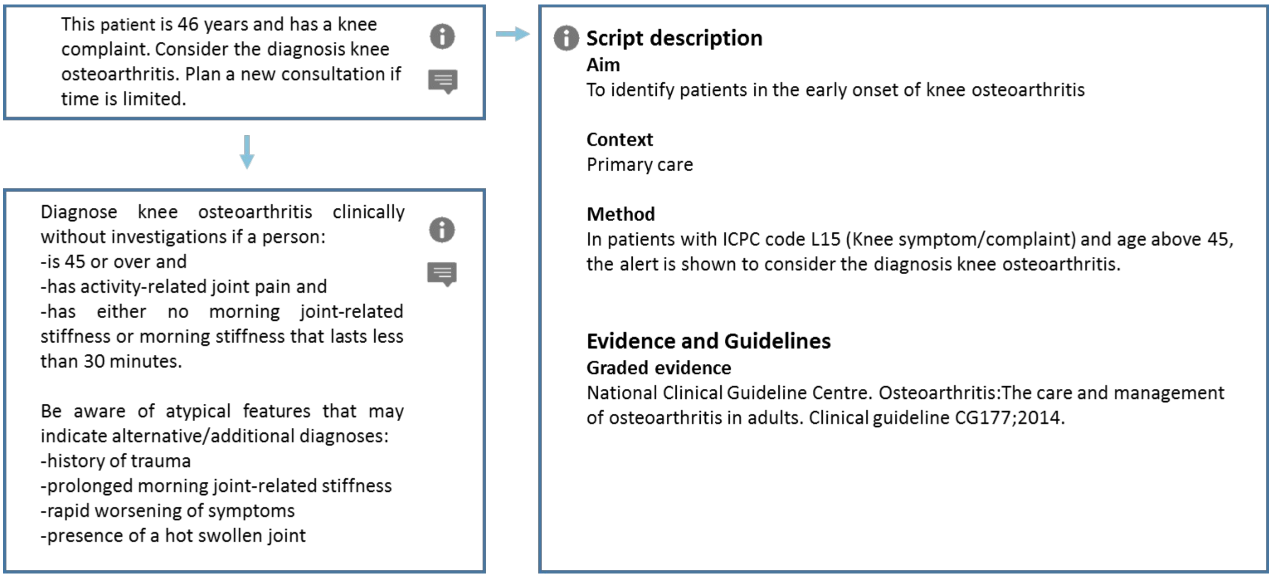

Figure 2: Illustration of the long reminder text for the diagnostic criteria that is available for the GP after clicking on the short reminder text and the justification for the reminder that is available after clicking on the information icon.

The doctor examines the patient and diagnoses it as knee osteoarthritis. After entering this information in the electronic record, the decision support system presents additional reminders (figure 3). In addition, links to a guideline and practical tools on knee osteoarthritis are displayed in the EHR.

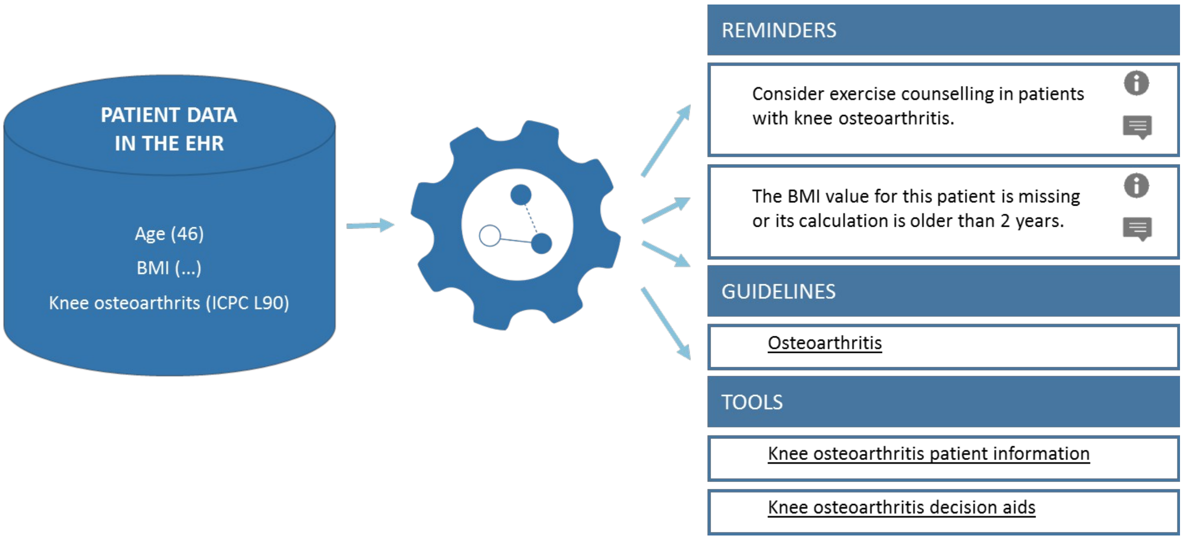

Figure3: Illustration of the generated computerised decision support in the style of EBMeDS after entering the diagnosis knee osteoarthritis. Paracetamol is available over the counter and often not registered in the electronic health record (EHR). This implies that the reminder can show up when actually paracetamol and topical NSAIDs have been used before oral NSAIDs. The BMI is not known for this patient.

Through reading the reminders, the doctor notices that no information on the patient's BMI is registered in the EMR. He measures the patient and enters a BMI of 24. This information deactivates the BMI reminder.

Next, the doctor and the patient discuss the benefits and practical issues for both supervised and unsupervised exercise. The doctor uses the link that is provided in the reminder to facilitate this discussion at the point of care. The tool is brief and can be used by the patient and the doctor together. Figures 4 presents the infographic for exercise. The doctor explains that on 100 patients like him, 55 patients would have benefits with exercise and that with usual care 30 patients would have benefits. This means that with exercise there are 25 patients more with benefits on 100 patients. This is also shown in a graph where the persons in black benefits, the persons in grey are those without benefits and the persons in red show the difference between exercise and usual care.

Figure 4: Illustration of the decision aid on exercise in the style of MAGICapp.

The patient also wants to know how much reduction in pain he can expect. The shows a new illustration and he explains that pain varies, but on average people that train will have 12 points less

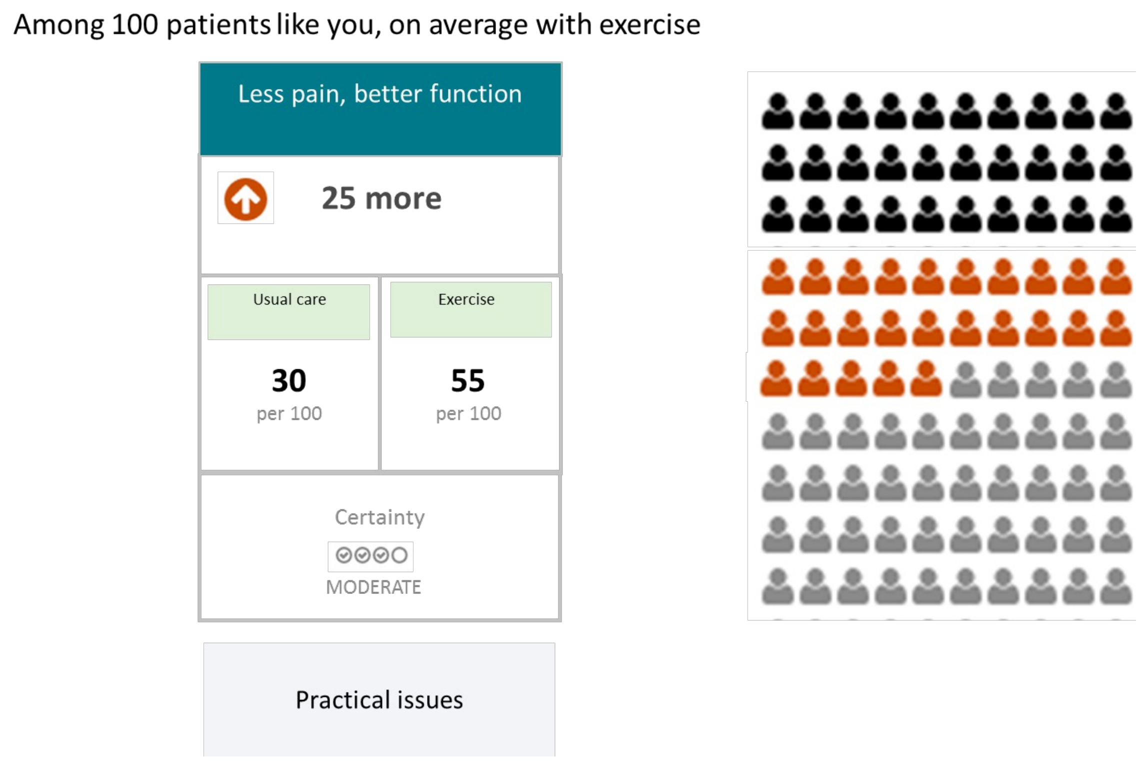

pain on a scale from 0 to 100.

.

Figure 5: Illustration of a second decision aid on exercise in the style of MAGICapp.

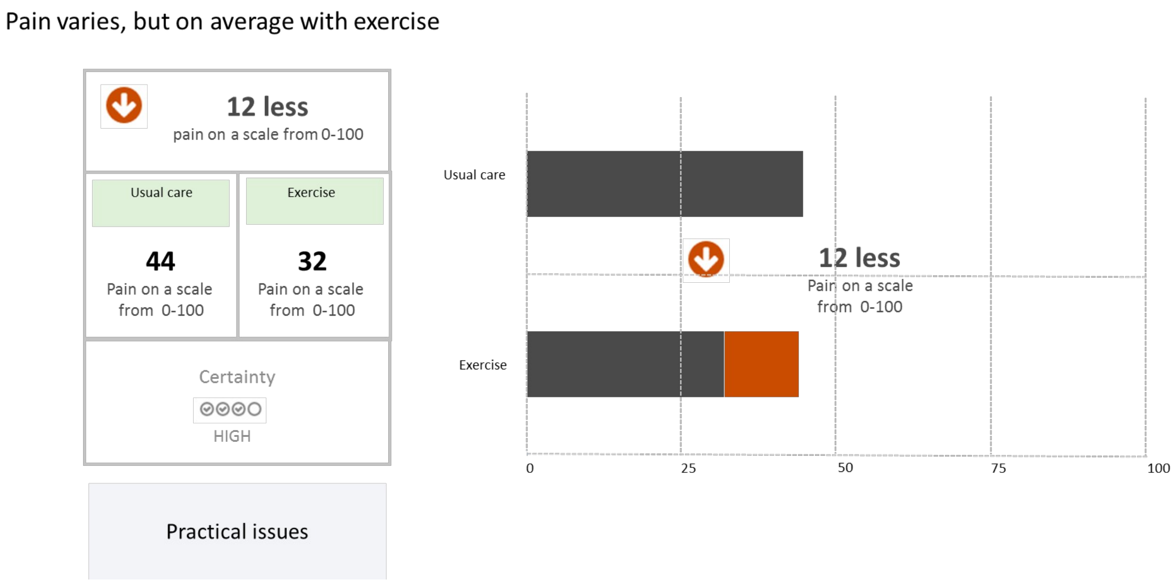

The data for these infographics is derived from data presented in the Cochrane review on exercise for osteoarthritis of the knee.[2]

However, the patient is worried about the side effects of exercises. The doctor clicks on the “Practical issues”-button.

Practical issues	
Procedure	May be performed supervised or unsupervised.<br>It requires motivation to exercise regularly. Some people find motivating themselves to do exercises hard.<br>Physiotherapy or group-based activities might form good alternatives.
Adverse effects	Generally, taking exercise is safe with few risks. Exercises may hurt at first and can cause some people to stop due to pain.<br>Starting slowly and increasing exercise gradually but surely reduces the risk of pain.<br>Non-weight-bearing exercises may be a good alternative e.g. cycling, swimming, aqua aerobics.

Figure 6: Illustration of the “practical issues” information in the decision aid in the style of MAGICapp.

After having discussed information, the patient is motivated to start bicycling again on a regular basis. The doctor de-activates the reminder on exercise counselling for the period of 1 year. The doctor also prescribes oral NSAIDs. The generates an additional reminder (figure ..). After reading the reminder, the GP advises the patient to continue taking the paracetamol painkillers.

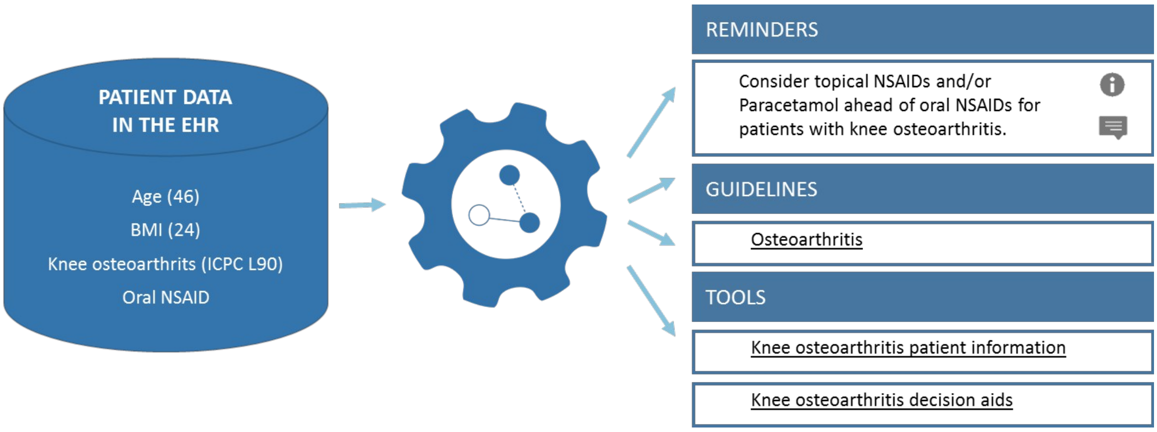

Figure 7: Illustration of the generated computerised decision support after having entered the BMI and after having prescribed an oral NSAID in the style of EBMeDS.

References

[1] Osteoarthritis: Care and Management in Adults. National Institute for Health and Clinical Excellence: Guidance. London2014.
[2] Fransen M, McConnell S, Harmer AR, Van der Esch M, Simic M, Bennell KL. Exercise for osteoarthritis of the knee: a Cochrane systematic review. Br J Sports Med. 2015;49(24):1554-7.
