# Supplementary material for: Development of a Tailored Intervention With Computerized Clinical Decision Support to Improve Quality of Care for Patients With Knee Osteoarthritis: Multi-Method Study
Source: JMIR Res Protoc. 2018 Jun 11;7(6):e154. doi: 10.2196/resprot.9927 (PMC6018233; doi:10.2196/resprot.9927)
Supplement: Multimedia Appendix 3 [file resprot_v7i6e154_app3.pdf]

short overview of the

GUIDES CHECKLIST

The GUIDES checklist provides an overview of success factors for guideline-based CDS and supports professionals to reflect over these factors in a structured way.

The website www.guidesproject.org provides access to an electronic version that enables CDS implementation teams to complete the GUIDES checklist efficiently in a group.

Domain 1: CDS context

1.1 CDS can achieve the defined quality objectives

1.2 The quality of the patient data is adequate

1.3 Stakeholders and users accept CDS

1.4 CDS can be added to the existing workload, workflows and systems

Domain 2: CDS content

2.1 The content provides trustworthy evidence-based information

2.2 The decision support is relevant and accurate

2.3 The decision support provides an appropriate call to action

2.4 The amount of decision support is manageable for the target user

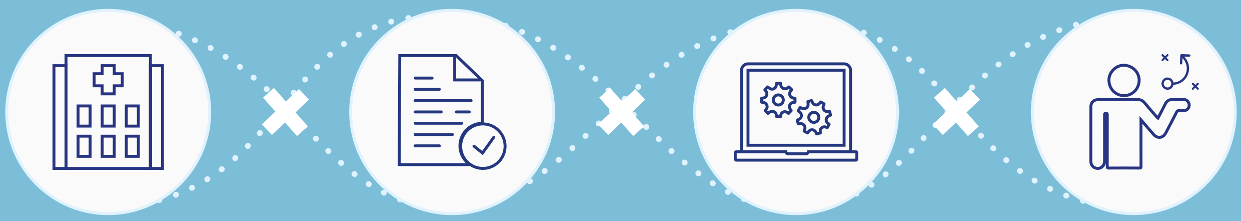

4 Domains that impact on the success of CDS to implement recommendations.

This project was headed by the Norwegian Institute of Public Health and has received funding from the EU's Horizon 2020 research and innovation programme.

Domain 3: CDS system

3.1 The system is easy to use

3.2 The decision support is well delivered

3.3 The system delivers the decision support to the right target person

3.4 The decision support is available at the right time

Domain 4: CDS implementation

4.1 Information to users about the CDS system and its functions is appropriate

4.2 Other barriers and facilitators to compliance with the decision support advice are assessed/addressed

4.3 Implementation is stepwise and the improvements in the CDS system are continuous

4.4 Governance of the CDS implementation is appropriate

Norwegian Institute of Public Health
