# Supplementary material for: Development of a Tailored Intervention With Computerized Clinical Decision Support to Improve Quality of Care for Patients With Knee Osteoarthritis: Multi-Method Study
Source: JMIR Res Protoc. 2018 Jun 11;7(6):e154. doi: 10.2196/resprot.9927 (PMC6018233; doi:10.2196/resprot.9927)
Supplement: Multimedia Appendix 4 [file resprot_v7i6e154_app4.pdf]

Additional file 4

Prioritisation of knee osteoarthritis recommendations

Recommendations	Summary of scores (for the overall assessment of the raters in Norway, Belgium and Finland)	Comments
**DIAGNOSIS**		
Diagnose knee OA clinically without investigations if a person is 45 or over and has activity-related joint pain and has either no morning joint-related stiffness or morning stiffness that lasts no longer than 30 minutes.	Median 4, IQR 4-5, Range 2-5	Selected. The Finnish experts did not prioritise this recommendation.
Radiography should not routinely be used to diagnose knee OA	Median 4, IQR 2.5-5, Range 1-5	
Laboratory test should not routinely be used to diagnose knee OA	Median 3.5, IQR 3-4, Range 3-4	
MRI should not be used standardly for diagnosing knee OA	Median 4, IQR 4-4.5, Range 3-5	We did not select this recommendation, since it is a logical consequence of the selected recommendation on diagnosing knee OA clinically.
**NON-PHARMACOLOGICAL MANAGEMENT**		
**Patients with knee OA should be educated and receive self-management information or be referred to self-management programmes**	Median 5, IQR 4.5-5, Range 4-5	Selected
Patients with knee OA should be instructed in the use of thermal modalities	Median 1.5, IQR 1-2, Range 1-4	
Patients with knee OA who are overweight, should be advised to lose weight	Median 5, IQR 4-5, Range 4-5	Selected
Patients with knee OA should be advised to engage in low-impact aerobic exercise (land or water based)	Median 4, IQR 4-5, Range 4-5	Selected

Patients with knee OA should be advised to participate in endurance and/or strengthening exercises	Median 5, IQR 4-5, Range 3-5	Selected
Patients with knee OA should exercise supervised by a physical therapist with manual therapy	Median 3.5, IQR 3-4, Range 2-5	
Patients with knee OA should receive walking aids as needed	Median 3, IQR 2.5-3, Range2-4	
Patients with knee OA should be advised to wear appropriate footwear/insoles	Median 3, IQR 3-3.5, Range 2-4	
**PHARMACOLOGICAL MANAGEMENT**		
Acetaminophen paracetamol (<4 g/day) should be used as an initial analgesic	Median 2.5, IQR 2-4, Range 2-5	
Oral NSAID should be used after acetaminophen	Median 4, IQR 4-4, Range 3-5	Selected
Topical NSAID should be used as adjunctive and alternative	Median 4, IQR 4-4.5, Range 3-5	Selected
Topical capsaicin should be used as adjunct and alternative	Median 2, IQR 1-2, Range 1-3	Topical capsaicin is not available to knee osteoarthritis patients in Finland
Gastroprotection is needed for high-risk patients	Median 4, IQR 3.5-4, Range 3-5	Selected
Use of Tramadol for refractory symptoms	Median 3, IQR 2-3, Range 2-3	
Opioids should be used for cases refractory to other modalities	Median 3, IQR 2.5-4, Range 2-4	
Intraarticular injections with corticosteroids should be used in the treatment of knee OA in patients with effusions or other physical signs of local inflammation	Median 3, IQR 3-3.5, Range 2-4	
**SURGERY**		
Joint replacement is recommended for appropriate patients with knee OA	Median 4, IQR 3-4.5, Range	
Arthroscopy with debridement is not recommended for the management of	Median 4, IQR 4-5, Range 4-5	Selected

symptomatic knee OA.
