# Supplementary material for: Development of a Tailored Intervention With Computerized Clinical Decision Support to Improve Quality of Care for Patients With Knee Osteoarthritis: Multi-Method Study
Source: JMIR Res Protoc. 2018 Jun 11;7(6):e154. doi: 10.2196/resprot.9927 (PMC6018233; doi:10.2196/resprot.9927)
Supplement: Multimedia Appendix 5 [file resprot_v7i6e154_app5.pdf]

Additional file 5 - Focus group data

Country	Group	Identified by patient or GP	Determinant from interviews	Prioritised	Suggested heading for determinant	Checklist domain	Unique to participant profile	Unique factor to country
Belgium	Leuven 1	Patient	CDS is acceptable for patients	No	Acceptability of CDS for patients	CDS Context	Patients	Three countries
Belgium	Leuven 1	Patient	Decision support is a good alternative for the time limitations of a consultation	No	Acceptability of CDS for patients	CDS Context	Patients	Three countries
Norway	Oslo 1	Patient	There is variation in attitudes about CDS depending on previous experiences with technology	No	Acceptability of CDS for patients	CDS Context	Patients	Three countries
Finland	Tampere 1	Patient	Not everybody (physicians and/or patients) will use or accept CDS that is directed at patients	No	Acceptability of CDS for patients	CDS Context	Patients	Three countries
Finland	Tampere 2	Patient	Healthcare professionals and patients will accept the CDS system	No	Acceptability of CDS for patients	CDS Context	Patients	Three countries
Belgium	Leuven 2	GP	The system should not limit the physician in his/her treatment choices	No	Acceptability of CDS for physicians	CDS Context	GPs	Two countries
Belgium	Leuven 2	GP	The system should inform and alert but not critisise the physician	No	Acceptability of CDS for physicians	CDS Context	GPs	Two countries
Belgium	Leuven 2	GP	Decision support should be constructive	No	Acceptability of CDS for physicians	CDS Context	GPs	Two countries
Norway	Oslo 1	GP	The attitudes toward CDS depend on how CDS is implemented because it might be disturbing	No	Acceptability of CDS for physicians	CDS Context	GPs	Two countries
Belgium	Leuven 2	GP	There are data gaps in the patient's medical record	No	Accuracy and completeness of the available patient data	CDS Context	Mixed	Three countries
Belgium	Leuven 1	GP	The patient's medical record needs to be of good quality	Yes	Accuracy and completeness of the available patient data	CDS Context	Mixed	Three countries
Norway	Oslo 1	GP	The quality of the patient data is good enough for CDS on knee osteoarthritis, often symptoms are recorded instead of diagnosis	No	Accuracy and completeness of the available patient data	CDS Context	Mixed	Three countries
Norway	Oslo 2	GP	Quality of patient data can vary from one electronic record to another	No	Accuracy and completeness of the available patient data	CDS context	Mixed	Three countries
Norway	Oslo 2	GP	Data about diagnosis, medication, age, blood pressure can be used by CDS	No	Accuracy and completeness of the available patient data	CDS context	Mixed	Three countries

Finland	Tampere 2	Patient	Patient records need to be updated and patients need to have confidence that the data is reliable	yes	Accuracy and completeness of the available patient data	CDS context	Mixed	Three countries
Belgium	Leuven 2	GP	CDS should not overload the clinician, the information could also be provided in a dosed way over time	No	Amount of decision support for healthcare professionals	CDS Content	GPs	Two countries
Norway	Oslo 1	GP	CDS can become too much when a patient has multimorbidities, the content should be prioritised	No	Amount of decision support for healthcare professionals	CDS content	GPs	Two countries
Norway	Oslo 2	GP	When the information becomes too much, then you loose focus	Yes	Amount of decision support for healthcare professionals	CDS content	GPs	Two countries
Belgium	Leuven 2	GP	CDS should not overload the patient, too much information will lead to forgetting parts of it	Yes	Amount of decision support for patients	CDS Content	Mixed	Two countries
Belgium	Leuven 2	GP	Information for the patient should be dosed over time	No	Amount of decision support for patients	CDS Content	Mixed	Two countries
Belgium	Leuven 1	GP	If CDS provides information about every problem a patient has, than that can be too much for the patient and might lead to unhappy patients	No	Amount of decision support for patients	CDS Content	Mixed	Two countries
Finland	Tampere 2	Patient	Amount of advice is kept under control	Yes	Amount of decision support for pa	CDS content	Mixed	Two countries
Norway	Oslo 1	GP	Decision support should be specific on which type of training	No	Appropriate call to action	CDS content	Mixed	Two countries
Norway	Oslo 2	Patient	It is important that CDS is concrete. For example which types of training work best?	Yes	Appropriate call to action	CDS content	Mixed	Two countries
Finland	Tampere 1	Patient	The information should contain precise advice on what type of exercise/mediciation a patient needs	Yes	Appropriate call to action	CDS content	Mixed	Two countries
Finland	Tampere 1	Patient	Patients are informed about different treatment options and have the opportunity to receive specific treatments	Yes	Appropriate call to action	CDS content	Mixed	Two countries
Finland	Tampere 1	Patient	Advice is understandable and easy to use	Yes	Appropriate call to action	CDS content	Mixed	Two countries
Finland	Tampere 2	Patient	Advice is reliable and understandable and always activated	Yes	Appropriate call to action	CDS content	Mixed	Two countries
Finland	Tampere 2	Patient	It is possible to use/do what CDS recommends	Yes	Appropriate call to action	CDS Content	Mixed	Two countries

Belgium	Leuven 1	GP	CDS helps the physician to be vigilant and not to forget certain treatment options	Yes	Benefits on health professional awareness and vigilance	CDS Context	Mixed	Two countries
Norway	Oslo 1	GP	It would be interesting for the physician to receive information on which patients are coping bad with their disease	Yes	Benefits on health professional awareness and vigilance	CDS Context	Mixed	Two countries
Norway	Oslo 2	Patient	Maybe clinicians will be more attentive to osteoarthritis with this strategy	Yes	Benefits on health professional awareness and vigilance	CDS context	Mixed	Two countries
Belgium	Leuven 2	GP	It is very positive that such a system can identify and fill gaps in the patient's medical record	Yes	Benefits on quality of patient data	CDS Context	Mixed	Three countries
Norway	Oslo 2	GP	Use of CDS can potentially motivate physicians to define diagnosis more specific instead of registering symptoms only	No	Benefits on quality of patient data	CDS context	Mixed	Three countries
Finland	Tampere 1	Patient	Necessary information has to be recorded in the patient record and it is positive that CDS stimulates good quality of patient record data (e.g. BMI)	Yes	Benefits on quality of patient data	CDS context	Mixed	Three countries
Belgium	Leuven 2	GP	CDS that is targeted directly at the patient can reduce the time before a new consultation is planned	Yes	Benefits on timely care	CDS context	GPs	One country
Belgium	Leuven 2	GP	Sufficient financial support for the CDS intervention is required	No	Budget for the CDS implementation	CDS Implementation	GPs	One country
Norway	Oslo 1	GP	Is the CDS information relevant?	No	Clinical appropriateness	CDS content	GPs	One country
Norway	Oslo 1	GP	How many of the decision support messages are irrelevant?	No	Clinical appropriateness	CDS content	GPs	One country
Norway	Oslo 2	GP	It is important that decision support provides nuances that are specific to a patient and that can be too big of a challenge for some problems	Yes	Clinical appropriateness	CDS content	GPs	One country
Norway	Oslo 2	GP	Which decision support is triggered when a patient has multiple diagnoses?	No	Clinical appropriateness	CDS content	GPs	One country
Belgium	Leuven 1	Not clear	Collect patient feedback about CDS through the physicians	No	Collection of user feedback	CDS Implementati	GPs	Two countries

Norway	Oslo 2	GP	Monitoring is important for those that manage the project in order to improve the strategy	Yes	Collection of user feedback	CDS Implementation	GPs	Two countries
Norway	Oslo 2	GP	Other electronic initiatives provide the opportunity for users to provide feedback, this channel is being used and their are persons that are responsible to follow up on this feedback	No	Collection of user feedback	CDS Implementation	GPs	Two countries
Belgium	Leuven 2	GP	CDS should be short and understandable in an eyeblink	Yes	Concise information	CDS System	GPs	Two countries
Belgium	Leuven 2	GP	CDS should be available in a short text that is to the point. It should be possible to click the decision support when further information is desired.	Yes	Concise information	CDS System	GPs	Two countries
Norway	Oslo 2	GP	CDS content should be short with a limited number of expandable layers that provide additional information	No	Concise information	CDS system	GPs	Two countries
Belgium	Leuven 1	Patient	Physicians need to devote sufficient time to the patient during consultations in general	Yes	Contact between the physician and the patient	CDS Context	Mixed	Two countries
Norway	Oslo 1	GP	CDS can disturb the contact between the physician and the patient	No	Contact between the physician and the patient	CDS context	Mixed	Two countries
Norway	Oslo 2	GP	CDS can disturb the contact between the physician and the patient	Yes	Contact between the physician and the patient	CDS context	Mixed	Two countries
Belgium	Leuven 2	GP	The clinician should have control over the system	No	Control over the system	CDS System	GPs	Two countries
Belgium	Leuven 1	GP	CDS should be customisable	Not clear	Control over the system	CDS System	GPs	Two countries
Norway	Oslo 1	GP	The clinician should have control over the system	No	Control over the system	CDS system	GPs	Two countries
Norway	Oslo 2	GP	The physician should have control over the system	Yes	Control over the system	CDS system	GPs	Two countries
Belgium	Leuven 2	GP	The source of the decision support should be reliable and based on guidelines	Yes	Credibility of the information	CDS Content	Mixed	Three countries
Belgium	Leuven 1	Patient	Patient information is not always reliable	No	Credibility of the information	CDS Content	Mixed	Three countries
Norway	Oslo 1	Patient	It is difficult to know how credible information is as a patient, and the patient trusts the clinician	No	Credibility of the information	CDS content	Mixed	Three countries

Norway	Oslo 1	GP	The information should be evidence-based	No	Credibility of the information	CDS content	Mixed	Three countries
Norway	Oslo 1	GP	The level of evidence for each recommendation should be clear	No	Credibility of the information	CDS content	Mixed	Three countries
Norway	Oslo 1	GP	It is not always easy to know if information is evidence-based, this should be clear	Yes	Credibility of the information	CDS content	Mixed	Three countries
Finland	Tampere 1	Patient	The information is evidence-based	Yes	Credibility of the information	CDS content	Mixed	Three countries
Norway	Oslo 1	Patient	Patients do not have access in the EMR. Can the future electronic patient record be an instrument to provide decision support to patients?	Not clear	Delivery channel of decision support for patients	CDS system	Patients	Two countries
Finland	Tampere 1	Patient	Decision support should appear in all the communication channels that a patient uses	Yes	Delivery channel of decision suppo	CDS system	Patients	Two countries
Finland	Tampere 2	Patient	The system needs a continuous developing process	Yes	Delivery channel of decision suppo	CDS system	Patients	Two countries
Belgium	Leuven 2	Patient	Patients want to receive information through their clinician	Yes	Delivery of decision support for patients with healthcare professional as an intermediate	CDS System	Mixed	Two countries
Belgium	Leuven 1	Patient	Decision support for patients should be delivered through the physician	No	Delivery of decision support for patients with healthcare professional as an intermediate	CDS System	Mixed	Two countries
Belgium	Leuven 1	Patient	Some patients want to receive information directly from the system	Yes	Delivery of decision support for patients with healthcare professional as an intermediate	CDS System	Mixed	Two countries
Norway	Oslo 2	GP	CDS that sends information directly to patients can be unacceptable for some patients and physicians. An alternative approach is that the physician first gets an overview of CDS for the patient and then decides if this is relevant to provide to the patient as an electronic notice.	No	Delivery of decision support for patients with healthcare professional as an intermediate	CDS System	Mixed	Two countries
Belgium	Leuven 2	GP	The decision support should inform about the effect that can be expected	No	Details about benefits and harms	CDS Content	Mixed	Two countries

Belgium	Leuven 1	GP	Decision support information about effects are trivial facts	No	Details about benefits and harms	CDS Content	Mixed	Two countries
Norway	Oslo 1	Patient	The outcomes of treatment choices should be clear for patients, also the outcomes of not doing something should be clear	No	Details about benefits and harms	CDS content	Mixed	Two countries
Norway	Oslo 2	GP	The way that you present the decision support and effect estimates is very important	Yes	Details about benefits and harms	CDS Content	Mixed	Two countries
Norway	Oslo 2	GP	Quick access to hard facts with very precise effect estimate information is needed	No	Details about benefits and harms	CDS Content	Mixed	Two countries
Belgium	Leuven 2	Patient	The effect of treatment options can differ from patient to patient	Yes	Differential treatment effects accross subgroups of patients	CDS Content	Mixed	One country
Belgium	Leuven 1	GP	Patients are different and some treatment options might not be relevant for specific patients	Yes	Differential treatment effects accross subgroups of patients	CDS Content	Mixed	One country
Belgium	Leuven 1	GP	Within the EHR, physicians already need to click a lot. CDS requires additional clicks and I don't know if I am motivated to do make that additional effort.	No	Effort required to use the system	CDS System	Mixed	Three countries
Norway	Oslo 2	GP	Experience with the system will reduce the time that is required to use it	Yes	Effort required to use the system	CDS system	Mixed	Three countries
Norway	Oslo 2	GP	Navigation in the CDS system should be easy	No	Effort required to use the system	CDS system	Mixed	Three countries
Finland	Tampere 1	Patient	The system is easy to use	Yes	Effort required to use the system	CDS system	Mixed	Three countries
Finland	Tampere 2	Patient	Easy to use for both professionals and patients	Yes	Effort required to use the system	CDS system	Mixed	Three countries
Finland	Tampere 2	Patient	Advice is available easily	No	Effort required to use the system	CDS system	Mixed	Three countries
Norway	Oslo 2	GP	CDS can connect data and information that is available	No	General	CDS context	/	/
Finland	Tampere 1	Patient	CDS has the potential to improve quality of care	Yes	General	CDS context	/	/
Finland	Tampere 2	Patient	CDS can improve the quality of care in healthcare services	Yes	General	CDS context	/	/
Finland	Tampere 2	Patient	A system produces advice to the right persons	Yes	General	CDS system	/	/

Norway	Oslo 2	GP	The system should be perfect when it is launched	No	General	CDS Implementation	/	/
Finland	Tampere 1	Patient	The implementation needs good management	Yes	General	CDS implemer	/	/
Finland	Tampere 2	Patient	Implementation is managed and system use is monitored	Yes	General	CDS implemer	/	/
Belgium	Leuven 2	GP	CDS should be available without costs for the patient	No	Impact of CDS on treatment costs	CDS Context	Mixed	One country
Belgium	Leuven 2	Patient	Patients accept to pay more for a visit if that leads to higher quality treatment	No	Impact of CDS on treatment costs	CDS Context	Mixed	One country
Finland	Tampere 1	Patient	The way the CDS is developed is important, especially the feasibility to use it on a daily practice for patients and physicians	Yes	Impact on the workload	CDS Context	Mixed	Two countries
Belgium	Leuven 2	GP	Decision support should fit in the workflow so that it has no negative impact on the amount of patients seen by the clinician	Yes	Impact on the workload of healthcare professionals	CDS Context	Mixed	Two countries
Belgium	Leuven 2	GP	The clinician is time pressured during consultations and decision support create stress for the physician	No	Impact on the workload of healthcare professionals	CDS Context	Mixed	Two countries
Belgium	Leuven 2	GP	Time is limited during a consultation and it may be necessary to plan an additional consultation to discuss the decision support	Yes	Impact on the workload of healthcare professionals	CDS Context	Mixed	Two countries
Belgium	Leuven 2	GP	CDS can save time for the clinician when searching for information	Yes	Impact on the workload of healthcare professionals	CDS Context	Mixed	Two countries
Belgium	Leuven 1	GP	Time during a consultation is too short when a patient comes for multiple problems	Yes	Impact on the workload of healthcare professionals	CDS Context	Mixed	Two countries
Belgium	Leuven 1	GP	Physicians lack time	No	Impact on the workload of healthcare professionals	CDS context	Mixed	Two countries
Belgium	Leuven 1	GP	Decision support information can compete with other information sources	Not clear	Impact on use of other systems	CDS Context	GPs	One country
Norway	Oslo 2	Patient	Governance should be public	Yes	Independence and conflicts of interest	CDS Implementation	Mixed	One country

Norway	Oslo 2	GP	Governance can be private, but not financed by drug industry	No	Independence and conflicts of interest	CDS Implementation	Mixed	One country
Finland	Tampere 1	Patient	Patients and physicians need Information about the system beforehand	Yes	Information about CDS	CDS implemen	Mixed	Two countries
Finland	Tampere 2	Patient	Appropriate information about the system is given to users	No	Information about CDS	CDS implemen	Mixed	Two countries
Belgium	Leuven 2	GP	The mode of information to users about the CDS system can be diverse	No	Information about CDS for healthcare professionals	CDS Implementation	Mixed	Two countries
Belgium	Leuven 1	GP	Regular updates are needed that communicate new content	No	Information about CDS for healthcare professionals	CDS Implementation	Mixed	Two countries
Norway	Oslo 2	Patient	When information is sent automatically to patients, then it should be clear for the patient why he/she receives this information	No	Information about CDS for patients	CDS Implementation	Patients	Two countries
Belgium	Leuven 2	GP	Possibility to address knowledge gaps for a physician	Yes	Information needs for healthcare	CDS Context	Mixed	Three countries
Belgium	Leuven 2	GP	Useful to remain up to date as a physician	No	Information needs for healthcare	CDS Context	Mixed	Three countries
Belgium	Leuven 1	GP	Decision support is not needed if you know the guideline	No	Information needs for healthcare	CDS Context	Mixed	Three countries
Belgium	Leuven 1	GP	It is important to have information about new recommendations	No	Information needs for healthcare	CDS Context	Mixed	Three countries
Norway	Oslo 1	GP	It would be interesting to know how many persons with knee symptoms get osteoarthritis	Yes	Information needs for healthcare	CDS Context	Mixed	Three countries
Norway	Oslo 2	GP	It is not sufficient to disseminate guideline information only on the internet	No	Information needs for healthcare professionals	CDS context	Mixed	Three countries
Finland	Tampere 1	Patient	It is necessary that CDS advice becomes integrated in every healthcare setting (Occupational health care, Physiotherapy, etc) and not only for the general practitioner	Yes	Information needs for healthcare	CDS context	Mixed	Three countries

Finland	Tampere 1	Patient	It is necessary that CDS helps the healthcare professional to choose the right medicine/treatment to the patient situation	No	Information needs for healthcare	CDS context	Mixed	Three countries
Finland	Tampere 1	Patient	Advice to the right person and professional group that cares for the patient e.g. a physiotherapist needs more information about exercise	No	Information needs for healthcare	CDS context	Mixed	Three countries
Finland	Tampere 2	Patient	Use CDS in all healthcare settings (Primary care, Occupational health care…)	Yes	Information needs for healthcare	CDS context	Mixed	Three countries
Norway	Oslo 1	Patient	CDS could also provide advice on refering to other healthcare professionals	Yes	Interaction with other healthcare professionals	CDS context	Patients	One country
Belgium	Leuven 2	Patient	The physician makes the decisions because the patient lacks knowledge	No	Lack of patient involvement	CDS Implementation	Patients	One country
Belgium	Leuven 1	Patient	Patients do not always get the possibility to make treatment choices	Yes	Lack of patient involvement	CDS Implementation	Patients	One country
Norway	Oslo 1	Patient	Links could be included to specific organisations and support groups	No	Making it easy to follow the decision support recommendation	CDS content	Mixed	One country
Norway	Oslo 1	Patient	Links should be included to practical information sources (for example videos)	Yes	Making it easy to follow the decision support recommendation	CDS content	Mixed	One country
Norway	Oslo 2	GP	Hyperlinks make it easier for physicians to find information	No	Making it easy to follow the decision support recommendation	CDS content	Mixed	One country
Belgium	Leuven 2	GP	Examples illustrating the advantage of using CDS should be clearly communicated	No	Marketing approach	CDS Implementation	GPs	Two countries
Belgium	Leuven 2	GP	The use of CDS could be promoted through the patient when they ask if their physician is using such a system	No	Marketing approach	CDS Implementation	GPs	Two countries
Norway	Oslo 2	GP	The system should be marketed and the best strategy is to demonstrate success through the involvement of superusers or through demonstration in pilots	Yes	Marketing approach	CDS Implementation	GPs	Two countries

Belgium	Leuven 1	GP	Decision support should be short during the consultation and long after the consultation	Yes	Match with workflow	CDS context	GPs	Two countries
Norway	Oslo 2	GP	When the patient comes with a new complaint, then the diagnosis is not entered directly in the electronic record and this might be a challenge for good decision support	Not clear	Match with workflow	CDS context	GPs	Two countries
Norway	Oslo 2	GP	It should be possible to continue working when the CDS system sends information	Yes	Match with workflow	CDS context	GPs	Two countries
Finland	Tampere 1	Patient	Advice appears automatically in situations when it is needed and right way	Yes	Mode of delivery	CDS system	Patients	One country
Belgium	Leuven 2	GP	It should be possible to receive decision support only on demand	Yes	Mode of delivery for healthcare professionals	CDS System	GPs	One country
Belgium	Leuven 2	GP	The decision support should not be delivered as pop-ups	Yes	Mode of delivery for healthcare professionals	CDS System	GPs	One country
Belgium	Leuven 1	GP	Decision support should be available automatically	Not clear	Mode of delivery for healthcare professionals	CDS System	GPs	One country
Belgium	Leuven 2	GP	More data is needed from patient about what works	No	Need for patient data on treatment adherence and effect	CDS Context	Mixed	One country
Belgium	Leuven 2	GP	Feedback from the patient is needed about adherence to treatment and the effect of the treatment	No	Need for patient data on treatment adherence and effect	CDS Context	Mixed	One country
Belgium	Leuven 1	Patient	The physician does not always know which treatment the patient chooses	Yes	Need for patient data on treatment adherence and effect	CDS Context	Mixed	One country
Belgium	Leuven 2	GP	Decision support should be discussed in person between the clinician and the patient. A risk from CDS is that it replaces this personal contact and that decision making occurs over the computer.	No	Need for personal discussion about the decision support between the healthcare professional and patient	CDS System	Mixed	Three countries
Belgium	Leuven 2	GP	It is necessary to verify how the patient understood the information and how this relates to other information that the patient received from other sources	No	Need for personal discussion about the decision support between the healthcare professional and patient	CDS System	Mixed	Three countries

Belgium	Leuven 2	Patient	Decision support could create anxiety with patients and it is necessary that the clinician can frame the information appropriatly	Yes	Need for personal discussion about the decision support between the healthcare professional and patient	CDS System	Mixed	Three countries
Belgium	Leuven 1	GP	Preferrably CDS for patients is delivered during the consultation so that the physician can advise the patient in person on the treatment choice based on the decision support information	Yes	Need for personal discussion about the decision support between the healthcare professional and patient	CDS System	Mixed	Three countries
Belgium	Leuven 1	GP	Information directed at the patient without advise by the physician can lead to inappropriate management	Yes	Need for personal discussion about the decision support between the healthcare professional and patient	CDS System	Mixed	Three countries
Belgium	Leuven 1	Patient	Patients could become too focussed or anxious when they receive decision support directly	No	Need for personal discussion about the decision support between the healthcare professional and patient	CDS System	Mixed	Three countries
Norway	Oslo 1	Patient	Technology alone is not enough, a lot has to do with the personal contact between the patient and the physician and the physician needs to know the patient his/her perspective	Yes	Need for personal discussion about the decision support between the healthcare professional and patient	CDS System	Mixed	Three countries
Finland	Tampere 2	Patient	Physicians should assess the CDS and give treatment advice, that is suitable for that patient	Yes	Need for personal discussion about the decision support between the healthcare professional and patient	CDS System	Mixed	Three countries
Finland	Tampere 1	Patient	Obstacles have been considered and if possible also addressed	Yes	Other obstacles and barriers	CDS implemer	Patients	One country
Finland	Tampere 2	Patient	Obstacles and enablers have been assessed	No	Other obstacles and barriers	CDS implemer	Patients	One country
Belgium	Leuven 1	GP	The reason why a patient asks for a consultation is not always clear	No	Patient data about the reason for encounter	CDS Context	Mixed	Two countries
Belgium	Leuven 1	GP	Having information before the consultation about the reason for encounter would be useful to prepare the encounter and patients could be invited to communicate this when ordering an consultation	No	Patient data about the reason for encounter	CDS Context	Mixed	Two countries

Belgium	Leuven 1	Patient	Not every patient wants to share information about the reason for encounter because of confidentiality of the information	No	Patient data about the reason for encounter	CDS Context	Mixed	Two countries
Norway	Oslo 1	GP	More information about the agenda for the consult could be collected from the reason for encounter lists	Yes	Patient data about the reason for encounter	CDS context	Mixed	Two countries
Belgium	Leuven 2	Patient	Patients want to receive more information about the disease and its management and decision support is very useful for that purpose	No	Patient information needs and demands	CDS Context	Mixed	Three countries
Belgium	Leuven 2	GP	It is an advantage when reliable information can be send to the patient, and now healthcare professionals often have to use time to reassure patients that have read inappropriate information from unreliable sources	No	Patient information needs and demands	CDS Context	Mixed	Three countries
Belgium	Leuven 2	GP	Good information to patients is required for good adherence to the treatment	Yes	Patient information needs and demands	CDS Context	Mixed	Three countries
Belgium	Leuven 1	Patient	Patients do not get sufficient treatment information. It is especially useful for the patient to get an overview of the treatment options	Yes	Patient information needs and der	CDS Context	Mixed	Three countries
Belgium	Leuven 1	GP	Not every patient wants to receive information	No	Patient information needs and der	CDS Context	Mixed	Three countries
Belgium	Leuven 1	GP	The physician does not alway know if the patient wants additional information	No	Patient information needs and der	CDS Context	GPs	One country
Belgium	Leuven 1	GP	Posters and flyers in the waiting room could inform patients that they can ask their physician about patient directed treatment information	No	Patient information needs and der	CDS Context	GPs	One country
Norway	Oslo 1	Patient	Patients want to have an overview of all the treatment options	Yes	Patient information needs and der	CDS Context	Mixed	Three countries
Norway	Oslo 1	Patient	It would be interesting for patients to receive feedback so that they know if what they do as a patient is good enough	No	Patient information needs and der	CDS Context	Mixed	Three countries

Norway	Oslo 1	Patient	More information for patient is needed about how to live with osteoarthritis and electronic information is not enough (for example links to osteoarthritis schools for patients, links to support groups)	No	Patient information needs and der	CDS Context	Mixed	Three countries
Norway	Oslo 2	Patient	Patients need a place where they can find reliable information	Yes	Patient information needs and der	CDS Context	Mixed	Three countries
Norway	Oslo 2	Patient	Patients need information about the most important information sources	Yes	Patient information needs and der	CDS Context	Mixed	Three countries
Norway	Oslo 2	Patient	It should be clear for patients what makes information reliable	No	Patient information needs and der	CDS Context	Mixed	Three countries
Norway	Oslo 2	GP	The physician and the patient often need the same information	Yes	Patient information needs and der	CDS Context	Mixed	Three countries
Norway	Oslo 2	Patient	Information for patients is essential and comes currently often too late	Yes	Patient information needs and demands	CDS Context	Mixed	Three countries
Finland	Tampere 1	Patient	The information for patients should be enough but not to much either	Yes	Patient information needs and der	CDS Context	Mixed	Three countries
Belgium	Leuven 2	Patient	Loosing weight is not easy and other factors than knowledge about recommended lifestyle changes will influence the success	No	Patient motivation and behaviour	CDS Implementati on	Mixed	Two countries
Norway	Oslo 1	Patient	Having knowledge about what to do is very important but other factors that influence if decision support is followed vary among individual patients	No	Patient motivation and behaviour	CDS Implementati on	Mixed	Two countries
Norway	Oslo 1	GP	It is challenging to motivate those patients that are not motivated. Can CDS help to motivate those patients?	Yes	Patient motivation and behaviour	CDS Implementati on	Mixed	Two countries
Norway	Oslo 2	Patient	Physiotherapy may be needed for patients that need to be motivated	Yes	Patient motivation and behaviour	CDS Implementati on	Mixed	Two countries
Norway	Oslo 2	GP	Healthcare professionals focus on the reason for encounter, while the patient might expect that the physician is also following up on concerns from earlier problems	No	Patient needs and demands in relation to follow-up	CDS Context	GPs	One country

Belgium	Leuven 2	GP	Patients can participate more in the decision making when he is better informed	Yes	Patient needs and demands in relation to shared decision making	CDS Context	GPs	One country
Norway	Oslo 1	Patient	Who enters patient data?	Not clear	Person responsible for data entry	CDS context	Patients	One country
Belgium	Leuven 1	GP	It is necessary to have insight in how the decision support is triggered in case you have doubts about the appropriateness of decision support	No	Reasoning process	CDS Content	GPs	Two countries
Belgium	Leuven 1	GP	The decision support should be linked to a diagnosis	No	Reasoning process	CDS Content	GPs	Two countries
Norway	Oslo 2	GP	CDS is ok for problems that are specific	No	Reasoning process	CDS Content	GPs	Two countries
Norway	Oslo 2	GP	Diagnosis and treatment data are the most important elements to trigger relevant CDS	Yes	Reasoning process	CDS content	GPs	Two countries
Belgium	Leuven 2	GP	After a while you get used to the decision support and the effect of alerts weakens because you do not longer look at it	No	Reduced notice with time	CDS System	GPs	One country
Belgium	Leuven 1	GP	After a while you will no longer give attention to the information that you have read multiple times before: This includes the risk that you do not notice that new information is available	No	Reduced notice with time	CDS System	GPs	One country
Belgium	Leuven 1	GP	After a while you do not longer read the routine information as a physician	Yes	Reduced notice with time	CDS System	GPs	One country
Norway	Oslo 1	GP	CDS can direct the topic of the consultation away from the specific reason why the patient asked for a consultation	Yes	Relation beteween CDS and agenda for the consultation	CDS content	GPs	One country
Norway	Oslo 1	GP	Decision support can be disturbing if it is not relevant for the consultation	Yes	Relation beteween CDS and agenda for the consultation	CDS content	GPs	One country
Norway	Oslo 1	GP	Irrelevant decision support is disturbing when providing urgent care as a doctor on duty	No	Relation beteween CDS and agenda for the consultation	CDS content	GPs	One country

Norway	Oslo 1	GP	It would be interesting if the physician could indicate at the start of the consultation what is the agenda for the encounter and that decision support information is triggered accordingly	No	Relation beteween CDS and agenda for the consultation	CDS content	GPs	One country
Norway	Oslo 2	GP	CDS can diverge the focus of the consultation to the topics suggested by the CDS instead of the problem raised by the patient.	Not clear	Relation beteween CDS and agenda for the consultation	CDS content	GPs	One country
Norway	Oslo 2	GP	Decision support should be relevant for the reason for encounter and this requires that the system is connected to a data field for the diagnosis specific for that day	No	Relation beteween CDS and agenda for the consultation	CDS content	GPs	One country
Belgium	Leuven 2	GP	The opinion of the clinician and the patient can be different	No	Role of personal opinions	CDS Implementati on	Mixed	Two countries
Norway	Oslo 1	Patient	Patients can have very strong beliefs and desires about which tests or treatment to receive	No	Role of personal opinions	CDS Implementati on	Mixed	Two countries
Norway	Oslo 2	Not clear	Patients and physicians can have beliefs and experiences that conflict with the decision support recommendations	No	Role of personal opinions	CDS Implementati on	Mixed	Two countries
Norway	Oslo 2	GP	CDS only becomes interesting if it covers a minimum number of problems	Yes	Scope of the decision support	CDS content	GPs	One country
Norway	Oslo 1	Patient	Detailed data are important but obtaining a lot of data can be a problem	Yes	Strategies to collect patient data	CDS context	Mixed	One country
Norway	Oslo 1	Patient	Strategies are needed to collect additional data to increase the potential for good decision support	No	Strategies to collect patient data	CDS context	Mixed	One country
Norway	Oslo 2	GP	Physicians should only be asked to enter additional patient data when this is realy important and having a positive impact on the patient outcomes	No	Strategies to collect patient data	CDS context	Mixed	One country
Belgium	Leuven 2	GP	Sufficient technical support is needed	No	Technical support	CDS Implementati on	GPs	One country

Belgium	Leuven 2	GP	It might be impossible for the clinician to read decision support before a consultation	No	Timing of decision support for healthcare professionals	CDS System	GPs	One country
Belgium	Leuven 2	GP	Presenting the decision support during the consultation is most effective	No	Timing of decision support for healthcare professionals	CDS System	GPs	One country
Belgium	Leuven 1	GP	When the reason for encounter is known, the decision support can be read before the consult.	No	Timing of decision support for healthcare professionals	CDS System	GPs	One country
Belgium	Leuven 2	Patient	Patients could receive information when making an appointment and this might motivate them to indicate the reason for their encounter	Yes	Timing of decision support for patients	CDS System	Patients	Three countries
Belgium	Leuven 1	Patient	It would be interesting for patients if they could have information available independently from a consultation. This makes it possible for patients to prepare themselves for a consultation with specific questions about treatment options that might apply to them	No	Timing of decision support for patients	CDS System	Patients	Three countries
Norway	Oslo 2	Patient	Patients need direct access to decision support so that they can prepare themselves for a consultation and therefore they need decision support information that is available before the encounter	Yes	Timing of decision support for patients	CDS system	Patients	Three countries
Finland	Tampere 1	Patient	CDS should come at the right moment	Yes	Timing of decision support for pati	CDS system	Patients	Three countries
Finland	Tampere 2	Patient	The system should work on the right moment	Yes	Timing of decision support for pati	CDS system	Patients	Three countries
Belgium	Leuven 2	GP	CDS needs to be intuitive, but training is always required	Yes	Training about CDS for healthcare professionals	CDS Implementati on	Mixed	Two countries
Belgium	Leuven 1	GP	Training is needed for the clinician to provide further background to CDS and to demonstrate how it functions	Yes	Training about CDS for healthcare professionals	CDS Implementati on	Mixed	Two countries
Norway	Oslo 1	Patient	CDS should be so intuitive so that it does not require any user training	No	Training about CDS for healthcare professionals	CDS Implementati on	Mixed	Two countries
Belgium	Leuven 2	GP	Decision support should be based on the most recent knowledge	No	Up to date information	CDS Content	Mixed	Two countries

Finland	Tampere 2	Patient	Updated advice (updated guidelines)	No	Up to date information	CDS content	Mixed	Two countries
Norway	Oslo 1	GP	If CDS is userfriendly then it might fit with the time limitations, there is no time to search for information during a patient encounter	Yes	Usability under time pressure	CDS system	GPs	Two countries
Norway	Oslo 2	GP	Time limitations is a challenge and it is important that CDS is well integrated	No	Usability under time pressure	CDS system	GPs	Two countries
Norway	Oslo 2	GP	The system should work fast and work with minimal data traffic	No	Usability under time pressure	CDS system	GPs	Two countries
Belgium	Leuven 2	GP	Visual aspects of how the information is presented should be considered	Yes	Visual style	CDS System	GPs	Two countries
Norway	Oslo 1	GP	CDS should be intuitive, with less text and more illustrations	Yes	Visual style	CDS system	GPs	Two countries
Norway	Oslo 1	GP	Differentiate in the presentation of content	Yes	Visual style	CDS system	GPs	Two countries
